# Screening NLRP3 drug candidates in clinical development: lessons from existing and emerging technologies

**DOI:** 10.3389/fimmu.2024.1422249

**Published:** 2024-07-30

**Authors:** Isak W. Tengesdal, Migachelle Banks, Charles A. Dinarello, Carlo Marchetti

**Affiliations:** ^1^ Department of Medicine, University of Colorado Denver, Aurora, CO, United States; ^2^ Department of Research, Rocky Mountain Regional Veteran Affairs (VA) Medical Center, Aurora, CO, United States

**Keywords:** NLRP3, small molecule inhibitor, inflammasome, IL-1, screening method

## Abstract

Decades of evidence positioned IL-1β as a master regulatory cytokine in acute and chronic inflammatory diseases. Approved biologics aimed at inhibiting IL-1 signaling have shown efficacy but variable safety. More recently, targeting NLRP3 activation, an upstream mediator of IL-1β, has garnered the most attention. Aberrant NLRP3 activation has been demonstrated to participate in the progression of several pathological conditions from neurogenerative diseases to cardio-metabolic syndromes and cancer. Pharmacological and genetic strategies aimed to limit NLRP3 function have proven effective in many preclinical models of diseases. These evidences have lead to a significant effort in the generation and clinical testing of small orally active molecules that can target NLRP3. In this report, we discuss different properties of these molecules with translational potential and describe the technologies currently available to screen NLRP3 targeting molecules highlighting advantages and limitations of each method.

## Introduction

NLRP3 belongs to the nucleotide-binding oligomerization domain (NOD)-like receptor (NLR) family of intracellular pattern-recognition receptors that sense infections or cellular perturbations. Stimulation of NLRP3, similarly to other NLRs, leads to the formation of intracellular macromolecular complexes termed inflammasomes. The initial event in the inflammasome formation process is the oligomerization of NLRP3 through homotypic interactions between NACHT domains with ATPase activity. The enzymatic activity of NLRP3’s NACHT domain has been shown to be vital for NLRP3 self-association and function (1). Next, is the recruitment of the inflammasome adaptor protein ASC [apoptosis-associated speck-like protein containing a caspase activation and recruitment domain (CARD)] through the PYD–PYD domain interactions. CARD-CARD domain interactions between ASC and caspase-1 mediate self-cleavage and activation of the inflammasome effector molecule caspase-1, which is responsible for the processing of the biologically inactive pro-IL-1β and pro-IL-18 into their biological active forms (2). Although NLRP3 function is critical for the host defense against infections, dysregulated NLRP3 activity leads to tissue damage. Currently, significant efforts are placed on investigations of the role of NLRP3 in disease progression, especially in sterile conditions like Alzheimer’s, Parkinson’s, cardio-metabolic syndromes and cancer where chronic inflammation has proven to mediate disease progression. This interest in NLRP3 is exemplified by the remarkable increase in scientific publications centered on this protein. In the past 5 years (2019–2023) 13’602 articles have been published on NLRP3 compared to the 4’347 articles published between 2014-2018 and the 1’212 listed from 2009 and 2013. This is likely driven by the attractive prospects of targeting NLRP3 for therapeutic development with small orally active molecules. In fact, numerous NLRP3 inhibitors with different chemotypes have been described and tested. This article reviews properties of these molecules and comments on current technologies to design and screen for potential candidates.

## NLRP3 in health and disease

Activation of NLRP3 is triggered by a diverse set of endogenous and pathogen-associated stimuli. This large spectrum of activation signals posits NLRP3 as a prominent sensor of cellular and tissue homeostasis, thereby affording NLRP3 a crucial role in host defense and disease.

For example, NLRP3 activation is necessary for initiating immune responses required for resolving infections (3–8). On the other hand, NLRP3 activation promotes age-related chronic inflammatory diseases through production of IL-1β. Here, NLRP3 is activated through endogenous signals in a tissue specific manner in rheumatological, metabolic, neurodegenerative, cardiovascular diseases and cancer.

Evidence for the driving role of NLRP3 and NLRP3-mediated IL-1β production in the progression of cardiovascular disease was demonstrated in the CANTOS study, a randomized placebo-controlled and world-wide study in 10,067 patients with atherosclerosis. In this study, IL-1β neutralization with the anti-IL-1β antibody canakinumab reduced secondary cardiovascular events (9).

In preclinical models of neurogenerative diseases, such as Alzheimer and Parkinson’s it has been shown that NLRP3 activation deteriorates cognitive and motor function in mice (10–12). Interestingly, recent investigations suggested a dispensable role for NLRP3 and caspase-1 in mouse models of Alzheimer’s (13). As also discussed by the authors, experimental differences in the model used including strain and age of the animals at analysis could have impacted the different outcomes. Another important example for the contribution of NLRP3 in the progression of sterile inflammation is in cancer. A pathological role for NLRP3/IL-1β has been demonstrated in several cancer models by promoting immunosuppression, tumor progression and reduced survival (14). The role of NLRP3 in other sterile conditions has been more extensively reviewed elsewhere (15–17). The consequences of deleterious NLRP3 activation are best demonstrated from patients with gain-of-function mutations in NLRP3. Cryopyrin-associated periodic syndromes (CAPS) are autoinflammatory diseases characterized by recurrent episodes of fever and systemic inflammation. These rare inherited autosomal dominant mutations in NLRP3 diseases are grouped in three different clinical phenotypes: neonatal-onset multisystem inflammatory disease/chronic infantile neurologic cutaneous articular syndrome (NOMID/CINCA) being the most severe form, Muckle–Wells syndrome (MWS) the intermediate phenotype and familial cold autoinflammatory syndrome (FCAS) the mildest form (18). IL-1 inhibitory agents have been shown to be effective in managing the clinical symptoms of these conditions. At present, NLRP3 inhibitors are under investigation in these rare diseases (**Table 1**).

**Table 1:** NLRP3 inhibitors in clinical trials.

Compound	Developer	Disease indication	Route	Clinical trial ID	Clinical Stage	Structure
Dapansutrile / OLT1177®	Olatec Therapeutics	Acute Gout, Type 2 Diabetes, Melanoma, Parkinson's*	Oral	NCT05658575, NCT06047262, NCT04971499	Phase 2,3	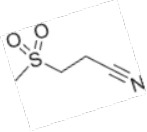
Dapansutrile / OLT1177®	Olatec Therapeutics	Osteoarthritis (OA)	Topical	NCT02104050, NCT01768975	Phase 2	
DFV-890	Novartis	OA, Familial Cold Auto-inflammatory Syndrome (FCAS), Coronary Heart Disease (CHD), CHD/Clonal Hematopoiesis of Indeterminate Potential (CHIP), Myeloid disesases, COVID-19/pneumonia	Oral	NCT04886258, EudraCT: 2020-006104-17, NCT04868968, EudraCT: 2020-005948-33, NCT06031844, CDFV890F12201, NCT06097663, NCT04382053, CADPT15A12201, NCT05552469, CDFV890G12101	Phase 2	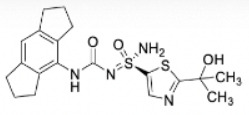
VTX-2735	Ventyx Biosciences	Cryopyrin-associated periodic syndromes (CAPS)	Oral	NCT04382053	Phase 2	NA
ZYIL1	Zydus Lifesciences	Safety, CAPS, Amyotrophic lateral sclerosis	Oral	NCT04731324, NCT04972188, NCT05186051, NCT05981040	Phase 1,2	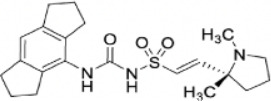
NT-0796	NodThera	Safety, Cardiovascular, Parkinson's	Oral	ACTRN12621001082897, NCT06129409, NA	Phase 1, 2	NA
VTX-3232	Ventyx Biosciences	Safety	Oral	NA	Phase 1	NA
Selnoflast RO-7486967	Roche	Parkinson's, CAPS, Asthma, CHD, Chronic Obstructive Pulmonary Disease (COPD)	Oral	NCT05924243, EudraCT/CTIS 2023-504412-14-00, ISRCTN73873157, EudraCT/CTIS 2023-504304-29-00, ISRCTN10520571, ISRCTN17672960, BP43098, EudraCT/CTIS 2021-000558-25	Phase 1	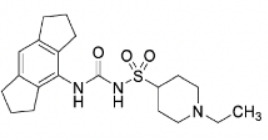
Emlenoflast / IZD174	Roche	CAPS	Oral	NCT04015076	Phase 1	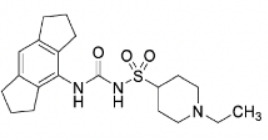
NNC6022-0001 (Vent-01)	Novo Nordisk	Safety	Oral	NCT06336005	Phase 1	NA
Vent-02	Ventus Therapeutics	Safety	Oral	NA	Phase 1	NA

*announced; NA, not available.

## Clinical development perspectives

The significant body of preclinical and clinical evidence of disease-promoting functions for NLRP3 and IL-1β has defined the rationale for testing NLRP3 inhibition in humans. NLRP3 inhibition presents several advantages compared to blocking IL-1 activity directly, most promising being the emergence of orally available NLRP3 inhibitors. Although numerous NLRP3 antagonist have been developed, no NLRP3 inhibitor has been approved in humans. As of today, several molecules are being tested in clinical trials. To the best of our knowledge, **Table 1** summarizes current clinical trials using specific NLRP3 inhibitors. Although these diseases have very different etiology, the NLRP3/IL-1 dependent inflammatory onset can be considered as common ground. Contrary to acute conditions, the efficacy of NLRP3 inhibition for the treatment of chronic diseases, such as metabolic, cardiovascular, cancer, and neurodegenerative disorders, will require treatment for long dosing duration. While no comparative clinical data are available yet, the pharmacology of the NLRP3 inhibitor class suggest to have increased safety compared to anti-IL-1 biologics. This would be due, in part, to increased specificity and shorter half-life. Increased specificity afforded by directly blocking NLRP3 would not prevent activation of the other members of the NOD family, therefore, the risk of opportunist infection reported with anti-IL-1 biologics should be more limited with NLRP3 inhibitors. Shorter half-life could be advantageous in scenarios where immunosuppression can be detrimental. Immune modulation becomes particularly relevant in patients with cancer or undergoing bone marrow transplant that are severe immunocompromised. Anti-IL-1 biologics, albeit extremely efficient, have a mean terminal half-life up to 26 days (19). Whereas, NLRP3 inhibitors have a significantly shorter half-life depending on the molecule, therefore, treatment can be interrupted without lingering effect. In clinical trials reported to date for VTX2735, developed by Ventyx and dapansutrile developed by Olatec, treatment has generally been well tolerated with minimal side effects (NCT05812781; NCT03534297). Common side effects included mild gastrointestinal symptoms and mild to moderate headaches. Since NLRP3 inhibitors suppress parts of the immune response, there is a theoretical risk of increased susceptibility to infections. However, specific data on increased infection rates in patients treated with NLRP3 inhibitors are not yet available. The long-term safety profile of these molecules is still being evaluated. Ongoing trials (**Table 1**) are expected to provide more detailed safety information over extended periods. Overall, while the safety profile of NLRP3 inhibitors appear favorable in early-stage trials, ongoing monitoring and larger studies are necessary to fully understand their long-term safety and potential adverse effects.

## Available technologies to screen NLRP3 inhibitors

Given the reported activity of NLRP3 in promoting the progression of several diseases, significant efforts have been devoted to developing effective NLRP3 inhibitors. This has led to a need for screening tests to evaluate potential candidates. Currently, the specificity and affinity of potential NLRP3 inhibitors can be tested in various ways. Here, we summarize and discuss the existing and recent advances in methodologies available for studying NLRP3 inhibitors.

### Cytokine measurement

Immunoassays determine the concentration of molecules in cell supernatants or bodily fluid. These techniques can be used to quantify the levels of cytokines, antibodies, antigens, proteins, lipids and hormones in body fluid or samples. The most common and versatile immunoassay is the enzyme-linked immunosorbent assay or ELISA. ELISAs are highly specific for measuring mature IL-1β and IL-18 following NLRP3 inflammasome induction, for example using LPS and ATP or LPS and nigericin stimulations. For samples limited by low volume or that require multiple cytokine readouts, multiplex systems allow multiple cytokine readouts with less than 50µl per sample. As of today, many commercially and user friendly available platforms have been developed. IL-1β and IL-18, as well as other cytokines, may also be assessed in real-time or living cells, by using the so called “Lumit” system (20). This immunoassay approach relies on labeled antibodies and a detention reagent that generate luminescent which can be detected in living cells or supernatants without requiring media removal or lysate transfer.

### Caspase-1 detection

Caspase-1 is the inflammasome effector enzyme responsible of the conversion of pro-IL-1β and pro-IL-18 into their respective biologically active forms. Therefore, a common measurement of inflammasome formation is detecting caspase-1 activity. Similar to IL-1β and IL-18, caspase-1 is originally synthesized as a precursor form which is activated by autocatalytic cleavage. The conversion of pro-caspase-1 into active caspase-1 can be determined by standard molecular technology like western blotting, measuring the levels of pro-caspase-1 (45KDa) and active caspase-1 (10 and 20KDa). Caspase-1 activation can be also measured by the addition to the testing system of a specific caspase-1 substrate, that once cleaved, releases measurable luminescence (21).

### ASC specks

Following activation of NLRP3, recruitment of the inflammasome adaptor protein apoptosis-associated speck-like protein containing a CARD (ASC) is initiated. ASC self-associates, forming the so-called ASC specks or pyroptosome that can be used a readout for inflammasome formation (22–24). While the intracellular formation of ASC specks has been well described and documented, more recently it has been demonstrated that these structures can be also be detected extracellularly (25). This is particularly relevant in the context of clinical trials due to the increased feasibility of measuring ASC specks in plasma and cerebrospinal fluid rather than tissues.

### Fluorescent resonance energy transfer

Another way of determining inflammasome formation is the determination of the association of the inflammasome sensor with the adaptor and/or the effector protein. A common technique used to achieve this goal is fluorescent resonance energy transfer (FRET) analysis. This technology permits the determination of the proximity between two molecules, for example NLRP3 with ASC and/or NLRP3 with caspase-1, within several nanometers, a distance sufficiently close for molecular interactions to occur. Typical fluorescence microscopy techniques rely upon the absorption by a fluorophore of light at one wavelength (excitation), followed by the subsequent emission of secondary fluorescence at a longer wavelength. Fluorescence resonance energy transfer is a process by which radiationless transfer of energy occurs from an excited state fluorophore to a second chromophore in close proximity. Because the range over which the energy transfer can take place is limited to approximately 10 nanometers (100 angstroms), and the efficiency of transfer is extremely sensitive to the separation distance between fluorophores, resonance energy transfer measurements can be a valuable tool for probing molecular interactions (26). Differently from measuring IL-1β and IL-18 processing, ASC specks formation and caspase-1 activity, FRET allows to determine directly the proximity of a specific inflammasome sensor with its adaptor protein increasing the specificity of the analysis.

### NLRP3-engament assay NanoBRET

Albeit the majority of the techniques described are reliable tools to measure inflammasome activity, they are not necessarily specific for the NLRP3 inflammasome considering that ASC specks formation and caspase-1-mediated processing of IL-1β and IL-18 shared pathways among different NOD sensors.

More recently, the NLRP3 NanoBRET target engagement (TE) assay has been described as a method to measure direct NLRP3 engagement (27). Briefly, to quantify NLRP3 target occupancy, this assay uses an NLRP3-Nanuluc construct transfected into HEK293 cells, and a fluorescent tracer based on the structure of MCC950, an NLRP3 inhibitor that binds directly to the NACHT domain of NLRP3 (28–30). MCC950 is the most used preclinical NLRP3 inhibitor at the present time. Numerous studies have demonstrated the efficacy of MCC950 in a wide range of NLRP3-mediated inflammatory diseases. Mechanistically, MCC950 binds to NLRP3 with high affinity (K_D_=224nM) (29). In their screening assay, *Teske et al.* confirmed that MCC950 is a specific and potent NLRP3 inhibitor. The authors also tested six other NLRP3 inhibitors and concluded that of the seven NLRP3 inhibitor tested, one (Cy09) showed only partial engagement, and two (OXSI-2 and OLT1177) failed to show direct antagonism. These three compounds (Cy09, OXSI-2 and OLT1177) have extensive investigations demonstrating their inhibition on NLRP3 activation and NLRP3 inflammasome formation. Of these three, we are particularly familiar with OLT1177^®^ (dapansutrile). Our group and other independent investigations have demonstrated that dapansutrile is a specific NLRP3 inhibitor. From the large body of evidence available, we emphasize that dapansutrile reduces NLRP3’s ATPase activity and directly binds NLRP3 with a K_D_ of 1.2µM (31, 32). Compared to MCC950, dapansutrile has a lower affinity binding (0.224µM vs 1.2µM respectively) impacting the degree of inhibition, as also shown by our group in direct comparisons between the two molecules (31, 33). Since the NLRP3 NanoBRET TE assay is based on MCC950, we question the relevance of the assay for non-sulfonylurea chemotype based NLRP3 inhibitors with lower affinity binding. In this regard, we emphasize that such differences in chemotype and lower affinity are not a limitation but rather a safety advantage for clinical use in humans. Contrary to MCC950, non-sulfonylurea compounds like dapansutrile has shown a remarkable safe clinical profile and is conducting large phase 2/3 trials (**Table 1**) (31, 34, 35). As previously discussed in this article, with the expanding examples of the pathological role of NLRP3 in several chronic diseases, there is a clear need for safe NLRP3 inhibitors.

**Figure 1:**
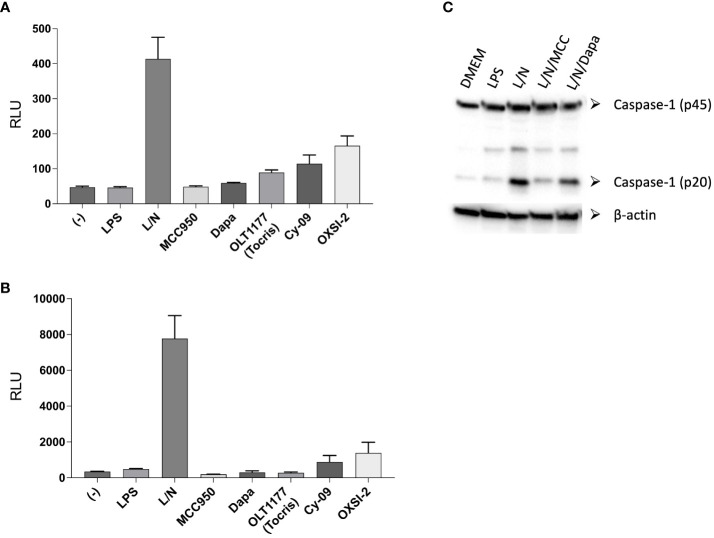
Lumit™ Mouse IL-1β Immunoassay under NLRP3 activating conditions. **(A, B)** J774A.1 cells were stimulated with LPS for 4 hours **(A)** or overnight **(B)** and nigericin for 60 minutes. NLRP3 inhibitors were added before nigericin. Lumit immunoassay was ran on whole cell culture as per manufacturer’s instructions. **(C)** Western blots for caspase-1 (p20 and p45) of cell lysates from J774A.1 cells stimulated with LPS and nigericin in the presence of MCC950 (MCC) and dapansutrile (Dapa).

Teske et al. added to their TE assay the testing of the inhibitors on IL-1β and caspase-1 processing. Similar to what they observed in the TE assay, the authors reported no activity for OXSI-2 and OLT1177 on the processing of IL-1β and caspase-1 in J774A.1 cells, a macrophage cell line commonly used to test NLRP3 activity. We measured IL-1β levels using the same detection assay used by the authors under canonical NLRP3 stimulation (LPS 4 hours at 1µg/ml and nigericin 10µM for 60 minutes) as well as priming the cells overnight (LPS 500ng/ml) as performed by the authors. Each of the NLRP3 inhibitors were tested at 10µM. Contrary to the data reported by Teske et al, both OXSI-2 and dapansutrile decreased IL-1β secretion as detected by the Lumit Mouse IL-1β Immunoassay (**Figures 1A, B**). Western blotting analysis exhibited a reduction in the levels of active caspase-1 (p20) by dapansutrile when compared to cells treated with LPS and nigericin (**Figure 1C**). Consistent with the *Teske et al*, and our previous data, MCC950 showed the strongest inhibition (**Figure 1**).

As a result, although we recognize the potential of the TE method under certain conditions, the proposed applications of the TE assay have significant limitations. Regarding the effect of OLT1177 and OXSI-2 on NLRP3 activity, we report data that confirmed a significant reduction in NLRP3 activity using the same conditions used by Teske et al. Moreover, for dapansutrile the large available body of clinical and preclinical evidence strongly support that dapansutrile is a specific NLRP3 inhibitor.

## Discussion

NLRP3 is emerging as an attractive therapeutic target to modulate the IL-1-mediated inflammation in several acute and chronic conditions. This is made possible by the development of specific NLRP3 inhibitors that present increased specificity compared to anti-IL-1 biologics and oral bioviability. To date, the most common chemotype for NLRP3 inhibitors is sulfonylurea compounds that have shown high potency and affinity binding but safety limitations. On the contrary, clinical and preclinical evidence suggest that non- sulfonylurea compounds present lower affinity but increased safety profile. Considering the reported role of NLRP3 in chronic conditions such as cancer, cardio-metabolic and neurodegenerative diseases, we believe this is particularly relevant considering the requirement of long-term treatment. With the increased interest in the development of effective and safe NLRP3 inhibitors, new specific and reliable screening tools are a clear need for the field. Albeit new technologies have been proposed (i.e. NLRP3 NanoBRET) they come with relevant caveats that could limit their use for non- sulfonylurea compounds. In this context, we recognize the need for additional technologies for functional and reliable screening assays for testing new anti-NLRP3 compounds entering this rapidly expanding field.

## Data availability statement

The raw data supporting the conclusions of this article will be made available by the authors, without undue reservation.

## Ethics statement

Ethical approval was not required for the studies on animals in accordance with the local legislation and institutional requirements because only commercially available established cell lines were used.

## Author contributions

IT: Investigation, Formal analysis, Data curation, Writing – review & editing, Writing – original draft, Methodology, Conceptualization. MB: Writing – review & editing, Methodology, Investigation, Formal analysis. CD: Writing – review & editing, Writing – original draft, Visualization, Supervision, Conceptualization. CM: Methodology, Writing – review & editing, Writing – original draft, Visualization, Supervision, Conceptualization.
